# Cardiac Diffusion Tensor Biomarkers of Chronic Infarction Based on In Vivo Data

**DOI:** 10.3390/app12073512

**Published:** 2022-03-30

**Authors:** Tanjib Rahman, Kévin Moulin, Luigi E. Perotti

**Affiliations:** 1 Department of Mechanical and Aerospace Engineering, University of Central Florida, Orlando, FL 32816, USA; 2 CREATIS Laboratory, Univ. Lyon, UJM-Saint-Etienne, INSA, CNRS UMR 5520, INSERM, 69100 Villeurbanne, France; 3 Department of Radiology, University Hospital Saint-Etienne, 42270 Saint-Priest-en-Jarez, France

**Keywords:** diffusion tensor imaging, in vivo cDTI, chronic infarction, cardiac microstructure, radial diffusivity, swine infarction model

## Abstract

In vivo cardiac diffusion tensor imaging (cDTI) data were acquired in swine subjects six to ten weeks post-myocardial infarction (MI) to identify microstructural-based biomarkers of MI. Diffusion tensor invariants, diffusion tensor eigenvalues, and radial diffusivity (RD) are evaluated in the infarct, border, and remote myocardium, and compared with extracellular volume fraction (ECV) and native T1 values. Additionally, to aid the interpretation of the experimental results, the diffusion of water molecules was numerically simulated as a function of ECV. Finally, findings based on in vivo measures were confirmed using higher-resolution and higher signal-to-noise data acquired ex vivo in the same subjects. Mean diffusivity, diffusion tensor eigenvalues, and RD increased in the infarct and border regions compared to remote myocardium, while fractional anisotropy decreased. Secondary (e_2_) and tertiary (e_3_) eigenvalues increased more significantly than the primary eigenvalue in the infarct and border regions. These findings were confirmed by the diffusion simulations. Although ECV presented the largest increase in infarct and border regions, e_2_, e_3_, and RD increased the most among non-contrast-based biomarkers. RD is of special interest as it summarizes the changes occurring in the radial direction and may be more robust than e_2_ or e_3_ alone.

## 1. Introduction

Cardiac function in health and disease depends on cardiac microstructure, which governs the preferential directions of contraction/relaxation and the mechanical/electrical properties [1,2] of the myocardium. Cardiac microstructure can be inferred from cardiac diffusion tensor imaging (cDTI), a magnetic resonance imaging (MRI) technique that allows the mapping of tissue microstructure in vivo without the use of contrast agents. Indeed, cDTI measures the intracellular and extracellular anisotropic diffusion of water molecules, from which the preferential orientation of cardiomyocytes and sheetlets is estimated [3,4].

As cDTI probes cardiac microstructure, it also provides information on microstructural changes occurring as a result of remodeling due to cardiac diseases, for example due to chronic myocardial infarction [5,6]. Remodeling due to scar formation post-myocardial infarction may lead to increased wall stress, reduced ejection fraction, and wall thinning, affecting the overall cardiac function [7]. Understanding the changes in cardiac microstructure due to myocardial infarction (MI) could provide valuable insight into the post-MI remodeling process.

MRI sequences such as T1-mapping and late gadolinium enhancement (LGE) are commonly used to identify the location and extent of the infarcted myocardium. These imaging sequences require the use of gadolinium-based contrast agents, which can have adverse effects on patients with pre-existing renal conditions [8].

Diffusion tensor invariants, such as mean diffusivity (MD) and fractional anisotropy (FA), can be used to detect and quantify the extent of MI with the additional benefit of providing insight into cardiac microstructure. In chronic infarcted tissue, previous studies [9–11] have reported an increase in MD and a decrease in FA, indicating overall microstructural changes post-MI. However, these overall microstructural changes have not carried over to specific changes in the preferential direction of the cardiomyocytes and along the sheetlets/cross-myofiber directions. Information regarding changes in these microstructural directions can be extracted from the diffusion tensor eigenvalues. Indeed, the primary eigenvalue (e_1_) corresponds to the diffusivity along the preferential direction of the cardiomyocytes [12], while the secondary (e_2_) and tertiary (e_3_) eigenvalues correspond to the diffusivity along the sheetlet and cross-myofiber directions [13].

The main goal of this study is to refine the analysis of cardiac microstructural changes by studying the individual diffusion tensor eigenvalues and by computing radial diffusivity, a marker of both sheetlet and cross-myofiber diffusivity, using in vivo cDTI data (radial diffusivity is defined as the average of the secondary and tertiary eigenvalues). These quantities are then compared against diffusion tensor invariants (e.g., MD, FA), native-T1, and extracellular volume fraction (ECV) to establish their sensitivity in identifying the infarcted tissue and its microstructural changes.

In the infarcted region, the extracellular volume fraction increases due to the death of cardiomyocytes. We hypothesize that this increased extracellular space will mainly occur in the radial direction of the remaining cardiomyocytes and replacement fibrosis, and will be reflected by a higher increase in e_2_, e_3_, and radial diffusivity compared to the increase in e_1_ and MD. This hypothesis is also motivated by our previous work [14] based on ex vivo, high-resolution, and high signal-to-noise ratio (SNR) data. Moreover, to further understand the results computed from experimental data, we simulate numerically the diffusion of water molecules. The computed diffusion quantities are then compared with experimental results as a function of ECV. We conclude our study by discussing the most effective diffusion quantities to detect and characterize the remote, border, and infarct regions and their microstructural interpretation.

## 2. Methods

### 2.1. Animal Model and Infarct Induction

Animal care during infarct induction, imaging, and all experimental procedures followed protocol #2015–124 approved by the Institutional Animal Care and Use Committee of the University of California, Los Angeles.

These experiments were part of a larger study, whose scope is to characterize myocardial structure and function [15], and to identify the material behavior of the passive myocardium [16]. As part of this study, an infarct model was created using female Yorkshire swine subjects. In vivo and/or ex vivo MRI data necessary for the current study was successfully acquired in seven (N = 7) subjects. All subjects (N = 7) were included in the ex vivo analysis. Two subjects were excluded from the in vivo analysis since: (1) one subject died during MRI acquisition before in vivo cDTI data could be acquired; and (2) one subject presented a right ventricle infarct that was not visible on in vivo cDTI data due to the poor in vivo cDTI quality in the right ventricle. The remaining five (N = 5) subjects were considered for in vivo analyses.

Before the beginning of the experimental procedures, the animal subjects had time to acclimate for at least one week. At the time of the MRI exam, the subjects’ body weight was 59.5 kg ± 6.6 kg (mean ± standard deviation). Myocardial infarction was induced under general anesthesia using microspheres. Ketamine (12.5 mg/kg) and midazolam (1 mg/kg) were injected intramuscularly to induce anesthesia. After induction, carprofen (4 mg/kg) and buprenorphine (0.02 mg/kg) were administered intramuscularly to provide pre-emptive analgesia. During MRI, anesthesia was maintained using isoflurane (1.5–2%) and Lactated Ringer’s solution was administered (2–5 mL/kg/h).

After accessing the femoral artery using a Seldinger technique, a balloon wedge pressure catheter (7 French) was inserted and guided to the aortic sinus using a metal guidewire under X-ray fluoroscopy. Subsequently, a micro guidewire (0.014 inches) was used to select a branch of the left circumflex (LCx) or left anterior descending (LAD) artery and a balloon catheter (1.5 mm diameter) was inserted and inflated prior of injecting a volume of microspheres (90 μm Polystyrene microspheres) equal to 2.5–3.0 mL. After one minute to avoid microsphere backflow, the balloon catheter was deflated and extracted. In vivo imaging was conducted six to ten weeks after infarct induction to allow for the formation of scar tissue. Additional details regarding the experimental procedure may be found in [17].

A 3T MRI scanner (Prisma, Siemens Healthineers, Erlangen, Germany) was used for in vivo and ex vivo imaging. In vivo imaging protocols included: late gadolinium enhancement (phase-sensitive inversion recovery sequence, TE/TR = 1.6 ms/876 ms; flip angle = 20°; spatial resolution = 1.33 × 1.33 × 8.0 mm^3^, *N*_avg_ = 1), T1-mapping (modified look-locker inversion recovery sequence with motion correction, TE/TR = 1.04 ms/280.69 ms; flip angle = 30°; spatial resolution = 1.77 × 1.77 × 8.0 mm^3^, *N*_avg_ = 1), and cDTI (M1M2-nulled motion-compensated waveform sequence [18], TE/TR = 59 ms/5000 ms; flip angle = 90°; spatial resolution = 2.0 × 2.0 × 8.0 mm^3^; b-values = 0 s/mm^2^ and 350 s/mm^2^; *N*_dir_ = 12; *N*_avg_ = 30).

**Figure 1.**
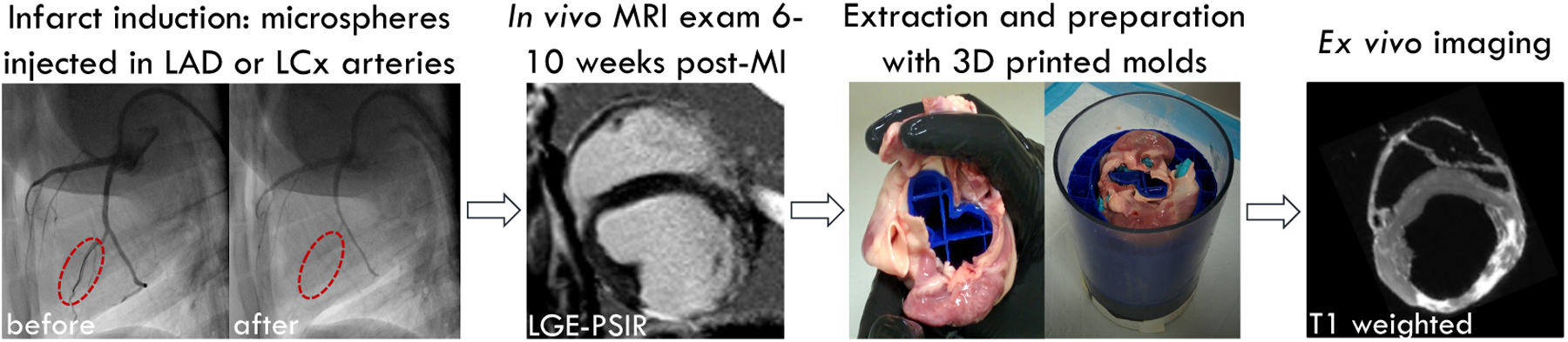
Experimental procedure: infarct induction and MR imaging

At the end of the in vivo MRI exam and before euthanasia, subjects were injected with a double dose of gadolinium-based contrast agent (0.6 mL/kg gadopentetate dimeglumine and 10 mL of saline solution). Euthanasia solution (0.1 mL/lb, Euthasol^®^, Virbac, Carros, France) was administered after 10 minutes to allow the circulation of the contrast agent. Hearts were then extracted, rinsed, and prepared for ex vivo imaging. To preserve ventricular geometry, the hearts were inserted into 3D-printed molds based on images acquired at mid-diastasis [19]. Subsequently, the hearts and molds were submersed in Fomblin perfluoropolyether (PFPE), and ex vivo imaging was conducted with a knee coil. On an average, ex vivo imaging began 2.5 h after euthanasia. A simplified flowchart summarizing the experimental procedure is shown in Figure 1.

Ex vivo imaging protocols included: T1-weighted GRE (TE/TR = 3.15 ms/12 ms; flip angle = 25°; acquisition matrix = 160 × 160; spatial resolution = 1.0 × 1.0 × 1.0 mm^3^, *N*_avg_ = 6), T2-weighted SE (TE/TR = 89 ms/15,460 ms; flip angle = 180°; acquisition matrix = 192 × 190; spatial resolution = 1.0 × 1.0 × 1.0 mm^3^, *N*_avg_ = 8), and cDTI (readout segmented sequence [20] with a twice-refocused spin-echo encoding [21], TE/TR = 62 ms/15,560 ms; acquisition matrix = 150 × 150; spatial resolution = 1.0 × 1.0 × 1.0 mm^3^; b-values = 0 s/mm^2^ and 1000 s/mm^2^; *N*_dir_ = 30; *N*_avg_ = 5).

### 2.2. Regional Subdivision and Registration

To quantitatively compare diffusion tensor quantities, Native T1, and ECV, the myocardium was subdivided into remote, border, and infarct regions. The regional subdivision process was based on late gadolinium enhanced (LGE) MR images.

Following the regional subdivision technique described by Schelbert et al. [22], regions of interest were drawn on the remote regions of each slice. Mean signal intensity (*μ*_remote_) and standard deviation (*σ*_remote_) were computed for each subject using regions of interest across all slices. Voxels with signal intensity (SI) less than the sum of mean signal intensity and two standard deviations were labeled as the remote zone (i.e., SI ≤ *μ*_remote_ + 2*σ*_remote_). To determine mean signal intensity (*μ*_infarct_) and standard deviation (*σ*_infarct_) of the infarct zone, small regions of interest (ROIs) were outlined on the most hyper-enhanced region of the remaining unlabeled myocardium of each slice. The mean signal intensities of remote and infarct zones were averaged to compute an intermediate value. Voxels with signal intensity higher than the sum of mean signal intensity and two standard deviations of the remote zone, but below the computed intermediate value (i.e., $\mu_{\text{remote}}\;+\;2\sigma_{\text{remote}}\;<\;\text{SI}\;<\;\frac{1}{2}\left(\mu_{\text{remote}}\;+\mu_{\text{infarct}}\;\right)$) were labeled as border zone. The remaining voxels (i.e., voxels with $\text{SI}\geq \;\frac{1}{2}\left(\mu_{\text{infarct}}\;+\mu_{\text{remote}}\;\right)$) were labeled as infarct zone. Based on this approach, label maps marking remote, border, and infarct regions were created for each slice.

**Figure 2.**
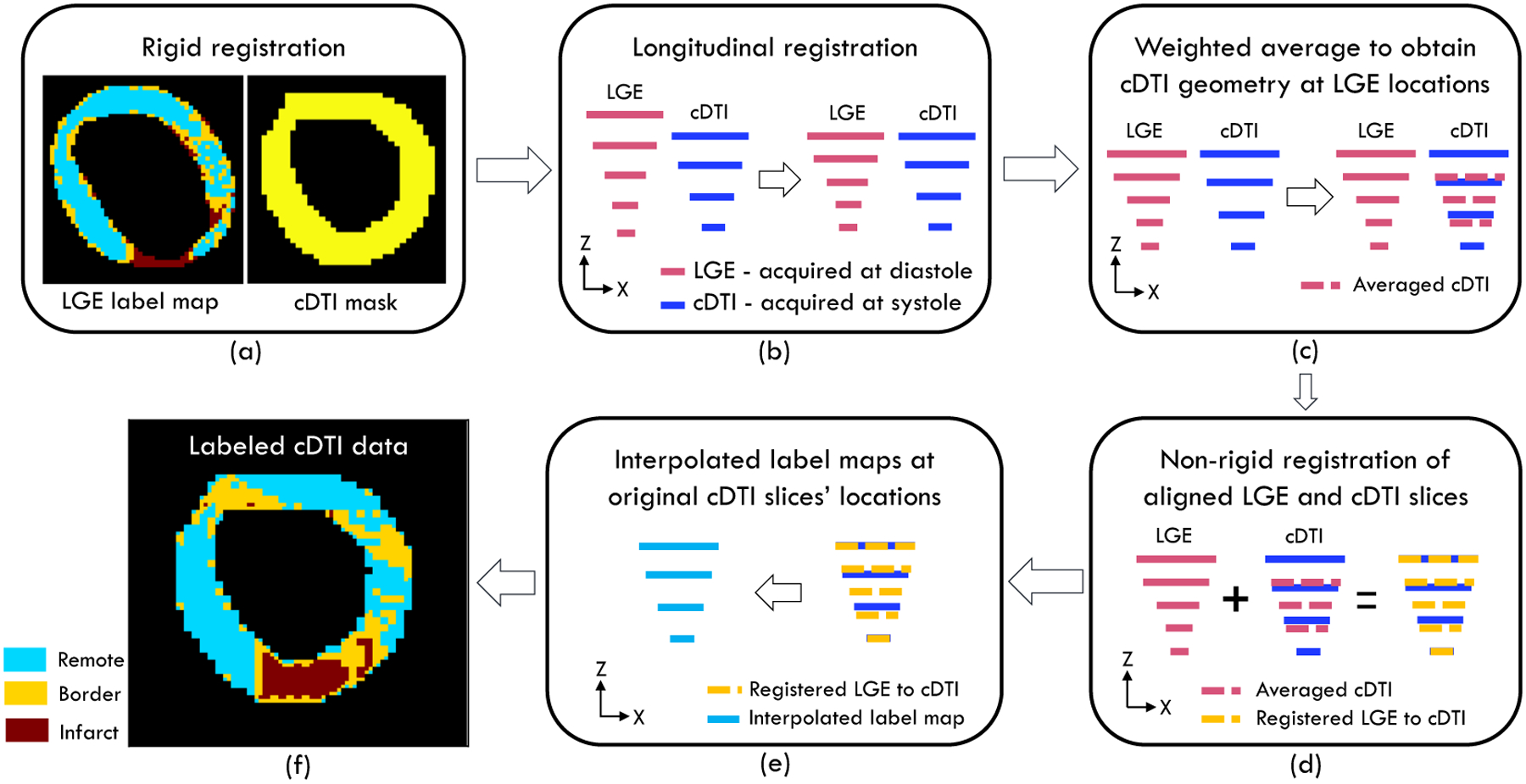
Diagram illustrating the registration process that was used to register LGE-based label maps to cDTI slices. (**a**) Quaternions were used to rigidly register LGE-based label maps to cDTI binary masks to ensure that both LGE and cDTI were in the same image space. (**b**) Since LGE was acquired during diastole and cDTI was acquired during systole, the longitudinal spacing between the LGE slices was reduced to reflect the contracted LV state during systole. This shortening was achieved by matching the basal and apical LGE and cDTI slices. (**c**) Although the basal and apical LGE and cDTI slices were aligned, the rest of the slices were not aligned. Hence, the cDTI slice masks at the *z*-axis location of the LGE slices were obtained via weighted average. (**d**) To obtain label maps at the cDTI location, aligned LGE-based label maps and weighted averaged cDTI slices were non-rigidly registered. (**e**) The cDTI label maps obtained in step (**d**) were at the *z*-axis location of the LGE slices. Hence, 3D interpolation was carried out to obtain label map values at the *z*-axis location of the original cDTI slices. (**f**) Based on the 3D interpolation, label maps for the remote (blue), border (yellow), and infarct (red) regions were obtained at the original cDTI slices’ locations.

LGE data were acquired during diastole while cDTI, due to its motion-compensated approach, was acquired during systole. Moreover, the LGE and cDTI sequences had different resolutions and fields of view. Hence, both rigid and non-rigid registrations were necessary to superimpose the LGE-based remote, border, and infarct zones to the cDTI data. For this purpose, quaternions [23] computed from LGE and cDTI images were used to rigidly register LGE nodes (each node corresponded to a voxel in the image space) to the cDTI nodes in the cDTI image space. This step was carried out to ensure that the LGE and cDTI nodes were in the same image space (Figure 2a).

To reflect the left ventricle (LV) longitudinal shortening during systole, spacing along the *z*-axis between LGE-based label map slices was reduced uniformly on a subject-specific basis. This was accomplished by matching the *z* coordinates of the most basal and apical LGE slices with the corresponding most basal and apical cDTI slices (Figure 2b).

At this stage, even though the basal and apical slices were at the same longitudinal locations, the rest of the slices were not aligned along the *z*-axis. Hence, a weighted average was carried out to obtain cDTI slices at the *z*-axis location of the LGE slices. First, for each subject and for each cDTI slice, the endocardium and epicardium borders were detected using the Canny edge detection method [24]. Then, at each LGE *z*-axis location, cDTI slices were calculated by averaging the cDTI endocardium and epicardium immediately above and below that *z*-axis location. The distances along the *z*-axis between the LGE-based label map slice and the cDTI slices immediately above and below were used as weights for this process (Figure 2c).

After obtaining all the cDTI slices at the LGE *z*-axis locations using the method described above, the LGE-based label maps were non-rigidly registered [25] to the cDTI slices at the LGE longitudinal locations. (Figure 2d).

Finally, the resultant LGE-based label maps were applied to the original cDTI slices via 3D interpolation (Figure 2e).

At the end of this registration process, each voxel in the original cDTI slices was labeled as either remote, border, or infarct as a function of the LGE-based maps (Figure 2f). Diffusion tensor invariants, eigenvalues (e_1_, e_2_, and e_3_), and radial diffusivity (RD) were associated with the remote, border, and infarct zones based on these maps.

### 2.3. Data Analysis

Diffusion tensor invariants and eigenvalues were computed after reconstructing the diffusion tensors from the acquired cDTI data. Voxelwise diffusion tensors were calculated using the freely available DiffusionRecon code [26] provided on GitHub and used in several previous studies (e.g., [4]).

ECV values at each voxel in the LV myocardium were computed from the subject Hematocrit, and from pre- and post-T1 mapping data according to [27].

To compare diffusion tensor invariants, eigenvalues, and ECV across remote, border, and infarct zones, all voxels in the LV myocardium were subdivided according to the registered LGE-based label maps. The ECV slices and cDTI slices were first rigidly and then non-rigidly registered according to the process described in Section 2.2; in this case, however, ECV slices were used instead of LGE label maps. On average, 2% (maximum 5.61%, minimum 0.61%) diffusion data were rejected due to having a mean diffusivity voxel value above the free diffusion of water (3 × 10^−3^ mm^2^/s).

Data are visualized using diffusion tensor quantities, ECV, and native T1 maps overlaid on five short-axis slices for one representative subject, and for all subjects using raincloud plots [28] and box plots across the remote, border, and infarct regions.

Remote, border, and infarct data for all subjects were initially examined for normality using the Anderson–Darling normality test. Since the data did not pass the normality test, the pairwise non-parametric Kruskal–Wallis test with Bonferroni post hoc adjustment was used to assess differences between remote, border, and infarct data across all subjects. The same pairwise test was also run for subject-wise remote, border, and infarct data. *p* < 0.01 was considered to be significant.

### 2.4. Numerical Modeling

Numerical simulations were carried out to investigate the relationship between ECV and diffusion tensor quantities. Tensor invariants and eigenvalues were computed from the simulated diffusion of particles, and ECV was computed based on the generated synthetic cell structures. Individual cardiomyocytes were represented by cylinders with 9 to 20 μm diameter and 100 μm length. Cells were then connected along the axial direction to form a cellular tree with branching added between trees. Cells were added in a 0.5 × 0.5 × 0.5 mm^3^ voxel until a target ECV was reached. Therefore, different ECVs corresponded to different cell densities and cell-to-cell distances. The displacements due to the diffusion of 20,000 water molecules were simulated in each voxel using a random walk approach [29] with a time step Δt of 10 μs for a total duration of 51 ms, which corresponds to the duration of the diffusion encoding in vivo [30]. The native diffusion coefficient D_0_ of the water molecules was 3 × 10^−3^ mm^2^/s and 2.2 × 10^−3^ mm^2^/s in the extra- and intracellular compartments, respectively [31]. The intracellular and extracellular compartments were kept impermeable.

The diffusion of water molecules resulted in an intra-voxel displacement distribution that was projected in 12 directions to mimic a diffusion tensor acquisition. Subsequently, the diffusion tensor corresponding to the simulated signal was reconstructed and its mean diffusivity, fractional anisotropy, eigenvalues, and radial diffusivity were calculated.

Five cell structures were generated per each target ECV. Nine target ECVs ranging from 20% to 100% were simulated, resulting in 45 different cell structures. For a given ECV and cell structure, each simulation was repeated five times, resulting in 225 diffusion simulations across ECVs and cell structures. To best approximate the simulated diffusion signal to the one acquired in the MRI experiment used in this work, only the extracellular water displacements were considered.

The code to perform the diffusion simulations described above is freely available on GitHub at [32].

## 3. Results

**Figure 3.**
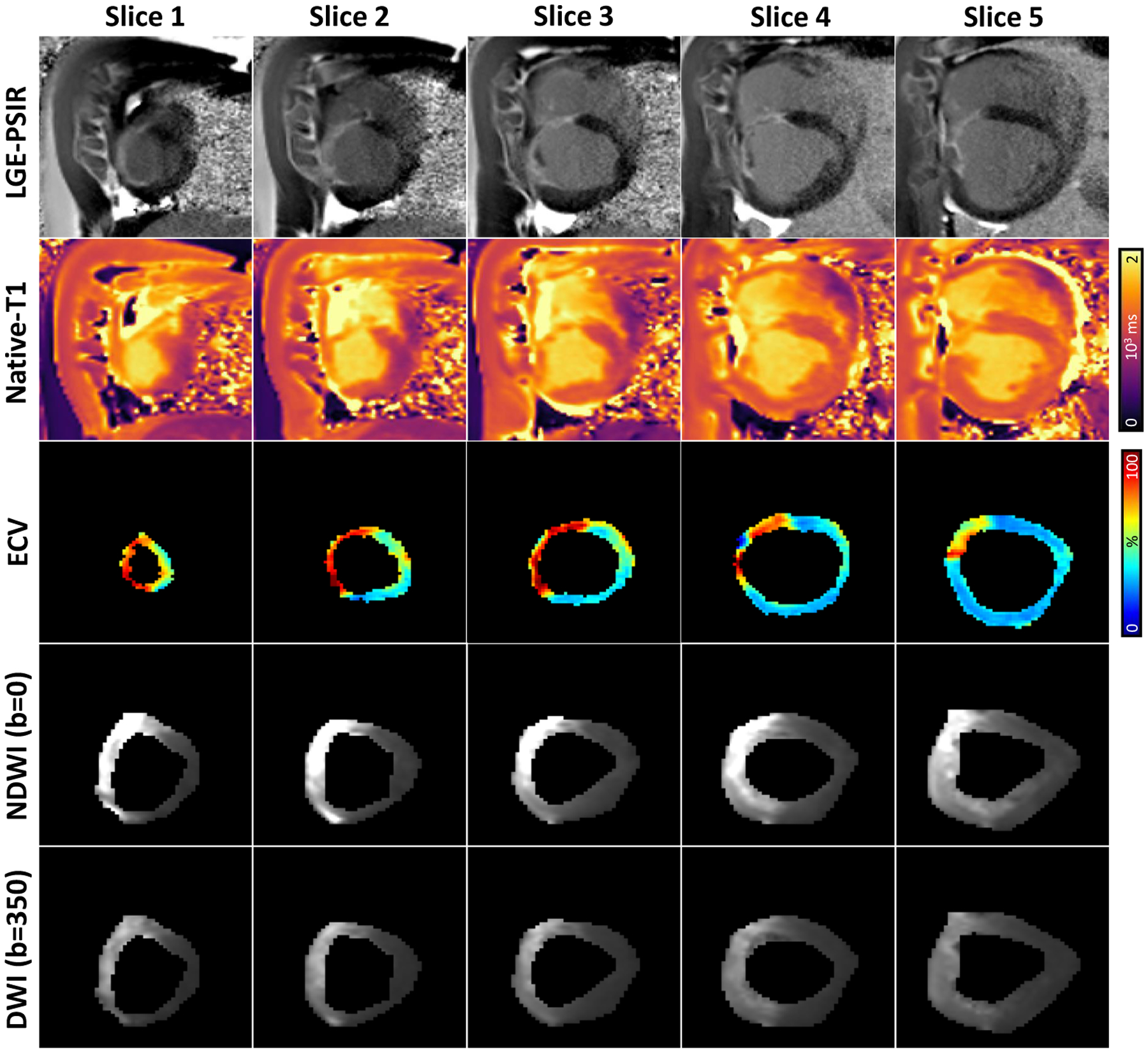
LGE-PSIR, native T1, ECV maps, b = 0 s/mm^2^, and b = 350 s/mm^2^ images for five representative short-axis slices of the same infarcted swine subject. LGE-PSIR is a contrast-based imaging technique, and native T1 and diffusion images (NDWI and DWI) are non-contrast-based techniques.

Figure 3 illustrates five short-axis representative slices of late gadolinium enhancement PSIR (LGE-PSIR), native T1, and the corresponding ECV maps along with b = 0 s/mm^2^ and b = 350 s/mm^2^ images of an infarcted swine heart.

**Figure 4.**
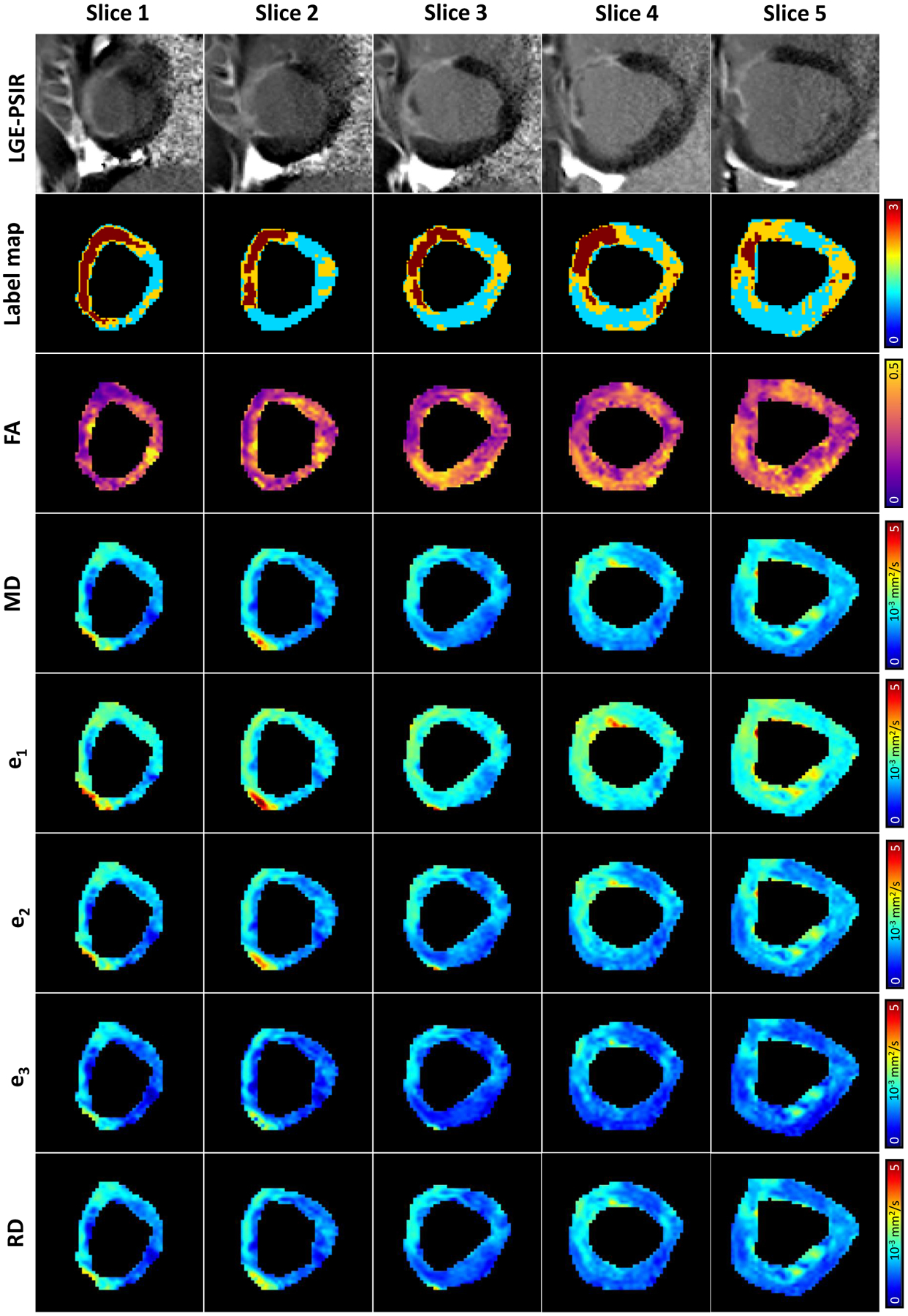
LGE-PSIR and corresponding label maps registered to cDTI along with fractional anisotropy (FA), mean diffusivity (MD), primary eigenvalue (e_1_), secondary eigenvalue (e_2_), tertiary eigenvalue (e_3_), and radial diffusivity (RD) maps for five representative short-axis slices in an infarcted subject.

LGE-PSIR images, along with their corresponding cDTI label maps and diffusion tensor quantities maps for the same five representative short-axis slices, are reported in Figure 4. As described in the Method section, the label maps were used to subdivide the corresponding FA, MD, e_1_, e_2_, e_3_, and RD maps in remote, border, and infarct regions. The border and infarct zones are distinguishable from the remote myocardium due to their markedly hyper-enhanced nature in the LGE-PSIR images. In the following tensor invariants and eigenvalue maps, the infarct and border zones exhibit a higher MD, e_1_, e_2_, e_3_, RD and a lower FA compared to the remote myocardium.

**Figure 5.**
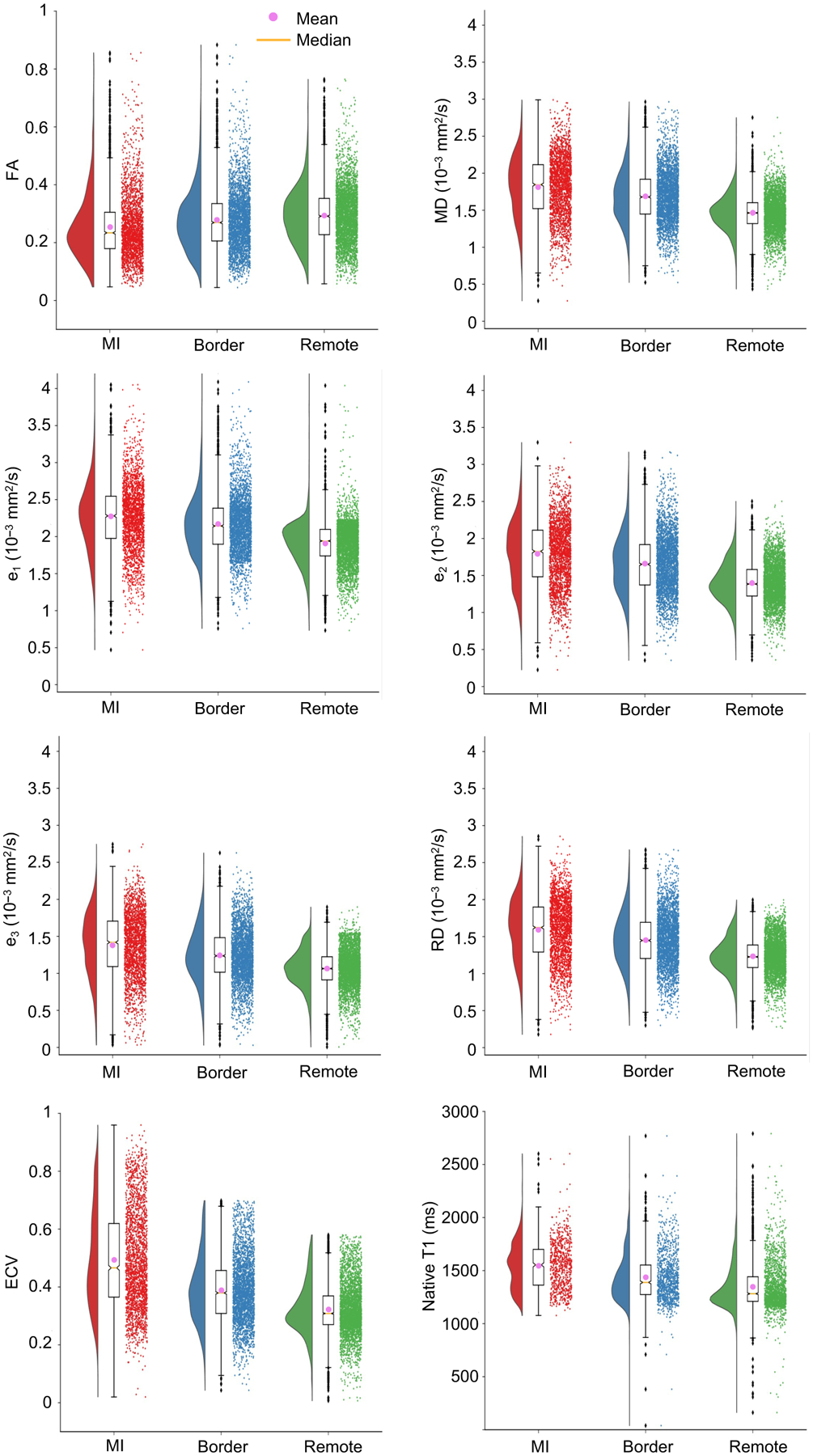
Raincloud plots [28] overlaid to the corresponding box plots for FA, MD, eigenvalues e_1_, e_2_, and e_3_, radial diffusivity (RD), extracellular volume fraction (ECV), and native T1 in the infarct, border, and remote myocardial regions.

In Figure 5, raincloud and box plots illustrate the distributions and quantitative differences of tensor invariants, eigenvalues, radial diffusivity, ECV, and native T1 across infarct, border, and remote regions. The 1st quartile (25th percentile) and the 3rd quartile (75th percentile) are marked by the lower and upper edges of the boxplot, respectively. The bottom and top whiskers mark the smallest and largest values within 1.5 times the interquartile range measured from the 25th and 75th percentiles, respectively. A summary of the mean, median, 1st, and 3rd quartiles over all subjects for all measured quantities are listed in Table 1.

Among native T1, diffusion tensor invariants, eigenvalues, and RD, the largest percent differences are observed for e_2_ (e_2_ increases by 19.2% and 31.9% from remote to border and infarct zones, respectively), e_3_ (e_3_ increases by 16.1% and 33.4% from remote to border and infarct zones, respectively), and RD (RD increases by 18% and 32.3% from remote to border and infarct zones, respectively). Overall percentage changes in median values for native T1, ECV, diffusion tensor invariants, eigenvalues, and RD between infarct, border, and remote regions are detailed in Table 2.

The computed *p* values were less than 0.01 for the statistical analyses carried out by grouping together the data across all subjects (Table 2), therefore showing that the observations in remote, border, and infarct regions did not originate from the same distribution. However, when the same statistical analyses were performed subject-wise, the resultant *p* values were greater than 0.01 in a few cases, especially in the native T1 distributions between infarct and border regions. All *p* values resulting from the pairwise non-parametric Kruskal–Wallis test for each subject are listed in Table 3.

**Figure 6.**
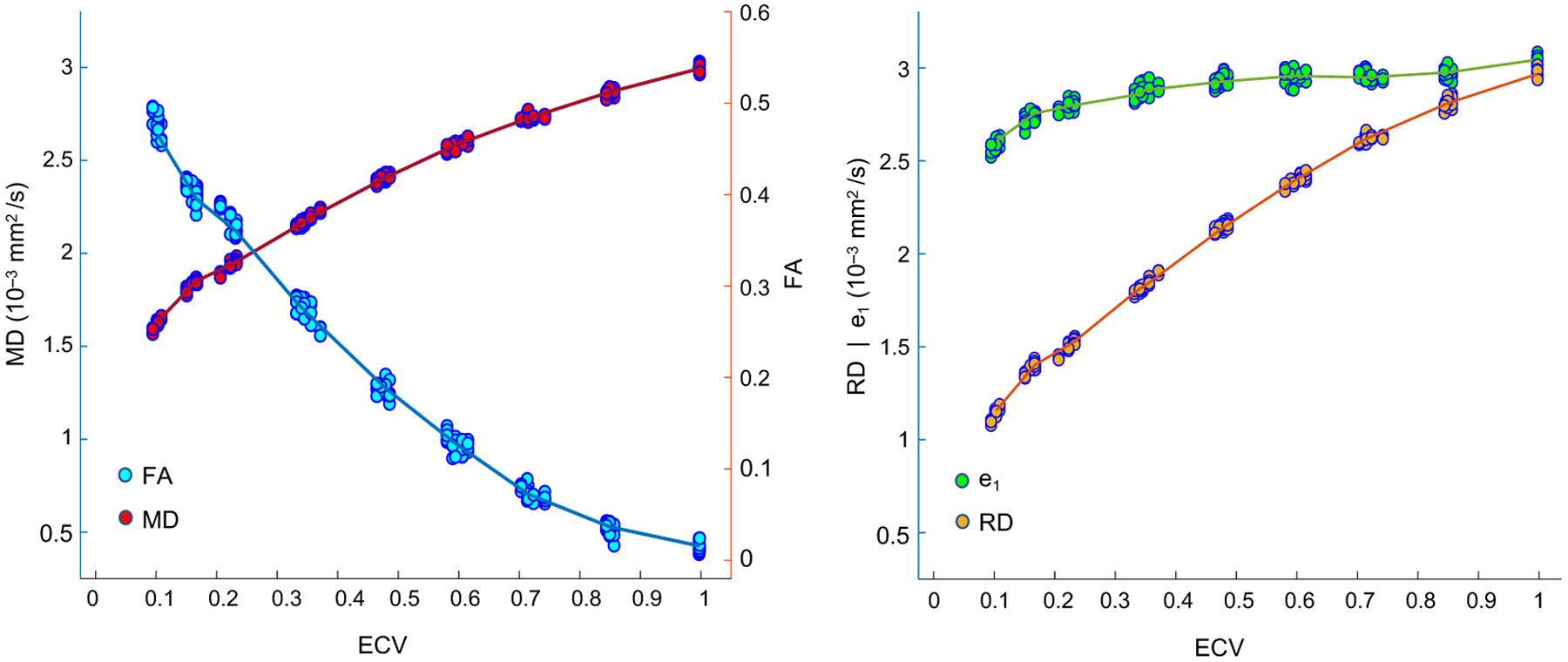
MD, FA, e_1_, and RD as a function of simulated ECV for 45 cellular structures and 5 diffusion distributions (225 cases in total). These results are obtained from diffusion simulations with 20,000 water molecules and a timestep Δt = 10 μs. Cellular structures with similar ECV (±0.05) are clustered together and median values of each cluster are connected to better visualize the overall trend of MD, FA, e_1_, and RD with increasing ECV.

The results of the diffusion simulations with 20,000 water molecules and a timestep Δt of 10 μs are illustrated in Figure 6. The change in MD, FA, primary eigenvalue (e_1_), and RD simulated as a function of ECV show that MD, e_1_, and RD increase with respect to ECV, while FA decreases. Furthermore, as ECV increases, the simulated percentage increase in RD is larger than the increase in e_1_.

**Figure 7.**
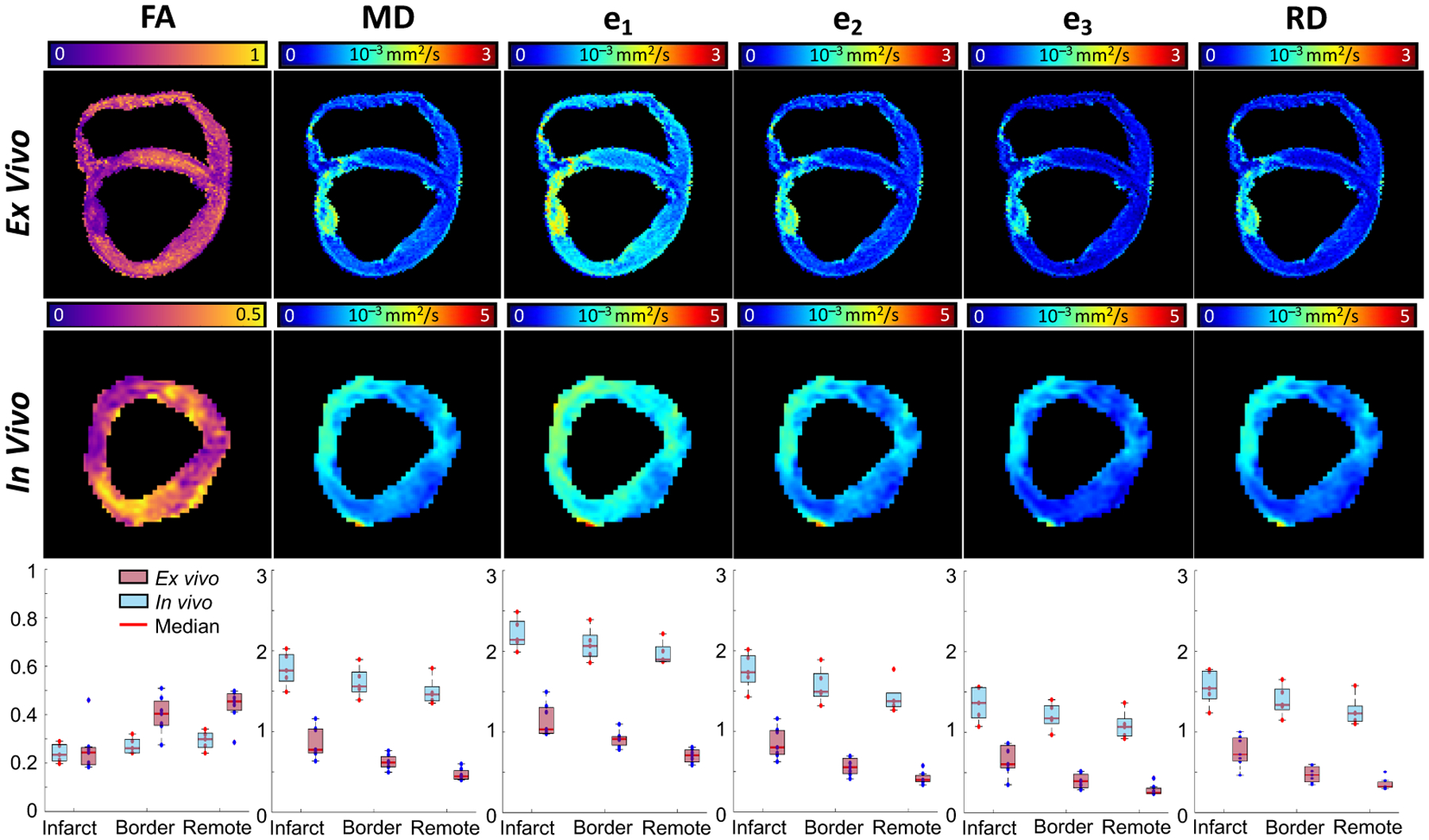
FA, MD, e_1_, e_2_, e_3_, and RD mappings for a representative slice from data acquired ex vivo (top) and in vivo (mid). Ex vivo and in vivo diffusion quantities across all subjects are compared using boxplots (bottom). These boxplots are constructed based on the subject-wise medians of the diffusion quantities.

Figure 7 compares in vivo and ex vivo FA, MD, e_1_, e_2_, e_3_, and RD values. Mappings are reported for a representative slice and boxplots were used to quantitatively compare the diffusion quantities computed using in vivo and ex vivo data across all subjects. The in vivo versus ex vivo boxplot comparisons are based on the subject-wise median values for FA, MD, e_1_, e_2_, e_3_, and RD.

## 4. Discussion

In this work, MRI data acquired in vivo in swine subjects with chronic MI were used to quantitatively analyze and compare ECV, native T1, diffusion tensor invariants, radial diffusivity, and eigenvalues across the remote, border, and infarcted myocardium. Among all analyzed quantities, RD, e_2_, e_3_, and ECV showed the highest percentage increase in the border and infarct regions with respect to the remote myocardium.

ECV has been identified as a potential biomarker to characterize affected myocardial tissue due to various cardiac diseases such as myocardial infarction [27] and hypertension [33]. After administration, Gadolinium-based contrast agents diffuse into the extracellular space. This causes the T1-relaxation time of the myocardium to depend on local gadolinium concentration [34]. The increased extracellular space in infarcted myocardium allows retention of a higher amount of gadolinium-based contrast. Hence, myocardial regions such as the border and infarct zones, where the extracellular space is higher due to the presence of replacement fibrosis, are expected to exhibit higher ECV compared to the remote myocardium [27]. The increased median ECV of the infarct (0.47) and border (0.38) regions compared to the remote (0.31) region observed in this work agrees with this mechanism.

Although widely used, ECV maps require the use of contrast agents, which are not indicated for patients suffering from pre-existing renal conditions. Among the existing non-contrast-based imaging techniques, native T1 has been considered to be an alternative biomarker for myocardial infarction detection [35], as well as for detecting myocardial edema and diffuse fibrosis [36–38]. In the current study, native T1 values in the infarct and border regions were 21.2% and 8.4% higher than the remote myocardium, respectively.

As with the case for native T1, diffusion tensor invariants and eigenvalues can also be computed without the use of contrast agents, and convey additional information regarding microstructural changes (e.g., myocardium anisotropy and inter-cellular spacing) occurring in the infarcted tissue. Among these quantities, FA decreased whereas MD, eigenvalues, and RD increased in the border and infarct regions when compared to the remote myocardium. Across all subjects, the percent increase or decrease of these parameters was higher in the infarct region compared to the border region. These trends concur with previous findings [9–11].

Chen et al. [39] validated histologically that after chronic myocardial infarction, the infarct region is largely comprised of replacement fibrosis, and the border region is a mixture of viable myocardium and replacement fibrosis. The presence of replacement fibrosis is reflected by an increased ECV. Due to the increase in extracellular space [40], the diffusion of water molecules increases. This is reflected by the increased MD values in the infarct and border regions computed in this study from experimental in vivo cDTI data. However, the observed increase in diffusion is not isotropic; instead, the increase in e_2_ and e_3_ is more significant than the increase in e_1_. This unequal increase in diffusion agrees with previous studies, suggesting that replacement fibrosis maintains the preferential direction of the replaced cardiomyocytes [41], and larger extracellular space is now present [40]. This corresponds to a larger diffusion increase in the e_2_ and e_3_ directions with respect to the increase in the e_1_ direction. The larger increase in the e_2_ and e_3_ directions concurs with a decrease in FA and agrees with histological findings [40].

As expected, due to a smaller amount of replacement fibrosis in the border zone, the increase in e_2_, e_3_, MD, and ECV and decrease in FA are less significant in the border region with respect to the infarct region.

To present diffusion values in the radial direction in a concise manner, radial diffusivity (RD) was used. RD is the average of e_2_ and e_3_ and represents the changes occurring in the radial direction due to the increase or decrease of ECV. In this study, we showed that RD increases significantly from the remote to the border (18% increase) and infarct (32.3% increase) regions.

The results computed from experimental data concur with the findings of particle diffusion simulations. From the diffusion simulations, as ECV increases, e_1_, e_2_, e_3_, and MD increase, and FA decreases. These trends agree with previous studies [42] and remain consistent regardless of the number of simulated water molecules (from four to thirty thousand water molecules per representative volume), length of adopted time step (from 10^−5^ s to 10^−7^ s), and cell structures (10 to 45 cell structures have been simulated). Additionally, as ECV increases, simulated e_2_ and e_3_ values increase at a higher rate with respect to e_1_.

Among T1-mapping, ECV and diffusion quantities, ECV demonstrated the highest change in the infarct (51.6% increase) and border (22.6% increase) regions compared to the remote myocardium. However, among the non-contrast-based methods, e_2_, e_3_, and RD exhibited the largest change. Moreover, the increases in e_2_ (31.9% and 19.2% increases from remote to infarct and border regions, respectively), e_3_ (33.4% and 16.1% increases from remote to infarct and border regions, respectively), and RD (32.3% and 18% increases from remote to infarct and border regions, respectively) were higher than native T1 (21.2% and 8.4% increases from remote to infarct and border regions, respectively).

In our previous study [14], ex vivo cDTI swine data acquired six to ten weeks post-infarction was used to compare diffusion tensor invariants, diffusion eigenvalues, and radial diffusivity across the remote, border, and infarct regions. In terms of the increase or decrease of tensor invariants, eigenvalues, and radial diffusivity in the infarct and border regions, similar trends were observed as in the present study (cf. Figure 7). However, while the diffusion eigenvalues and mean diffusivity computed using in vivo cDTI were observed to be higher than the values obtained using ex vivo data across all regions, their percentage changes were lower than the percentage changes computed using ex vivo data.

The median e_1_, e_2_, e_3_, and RD values computed using ex vivo data (values computed using in vivo data in this study are in parenthesis) were, respectively: 1.16 (2.28) × 10^−3^ mm^2^/s for e_1_, 0.88 (1.83) × 10^−3^ mm^2^/s for e_2_, 0.72 (1.42) × 10^−3^ mm^2^/s for e_3_, and 0.79 (1.62) × 10^−3^ mm^2^/s for RD, in the infarct region; 0.91 (2.14) × 10^−3^ mm^2^/s for e_1_, 0.55 (1.65) × 10^−3^ mm^2^/s for e_2_, 0.39 (1.24) × 10^−3^ mm^2^/s for e_3_, and 0.47 (1.45) × 10^−3^ mm^2^/s for RD, in the border region; 0.70 (1.94) × 10^−3^ mm^2^/s for e_1_, 0.41 (1.38) × 10^−3^ mm^2^/s for e_2_, 0.28 (1.06) × 10^−3^ mm^2^/s for e_3_, and 0.35 (1.22) × 10^−3^ mm^2^/s for RD, in the remote region.

The observed discrepancy between results obtained using in vivo and ex vivo data could be due to several factors, among which the in vivo motion artifact and perfusion. Furthermore, the MRI sequence parameters were not identical in vivo and ex vivo. The ex vivo data were acquired at a higher resolution of 1.0 × 1.0 × 1.0 mm^3^, higher SNR of ≈ 41 and higher b-value of 1000 s/mm^2^ while the in vivo data were acquired at a resolution of 2.0 × 2.0 × 2.0 mm^3^ with a SNR of ≈ 16 and b-value of 350 s/mm^2^. Higher resolution and SNR allow for a more accurate segmentation and subdivision of myocardium into remote, border, and infarct regions, therefore emphasizing the differences between regions.

Overall, the diffusion tensor invariants reported in this study are in good agreement with values reported in previous studies. Das et al. [43] conducted a study on 30 patients with myocardial infarction. The mean MD and FA values computed in vivo by Das et al., compared to the values computed in current study (reported in parenthesis), were: 1.83 × 10^−3^ (1.81 × 10^−3^) mm^2^/s for MD and 0.22 (0.25) for FA in the infarct region; 1.53 × 10^−3^ (1.69 × 10^−3^) mm^2^/s for MD and 0.33 (0.28) for FA in the border zone, and 1.45 × 10^−3^ (1.46 × 10^−3^) mm^2^/s for MD and 0.35 (0.29) for FA in the remote myocardium.

The computed mean ECV in Das et al. [43] was 0.60, 0.29, and 0.29 in the infarct, border, and remote regions, respectively, compared to 0.49, 0.39, and 0.32 in the current study. The discrepancies in the reported ECV values could be due to the different methods used to subdivide the myocardium into remote, border, and infarct regions. Moreover, Das et al. used in vivo human data, whereas in vivo swine data were used in the current study. The much larger cohort size (N = 30 vs. N = 5) compared to the current study could also be a reason for the observed discrepancies.

Stoeck et al. [9] conducted a study on five swine subjects with myocardial infarction. At nine weeks post-MI, reported mean MD and FA values in the infarct and remote zone were (values from the current study are reported in parenthesis): 1.66 × 10^−3^(1.81 × 10^−3^) mm^2^/s for MD and 0.28 (0.25) for FA in the infarct region; 1.34 × 10^−3^ (1.46 × 10^−3^) mm^2^/s for MD and 0.38 (0.29) for FA in the remote myocardium. Mean ECV values reported by Stoeck et al. were 0.83 and 0.31 in the infarct and remote regions, and in the current study, the mean ECV was 0.49 and 0.32, respectively. This discrepancy could be due to the uncertainty associated with native T1 scans and the computed ECV [44]. Differences in imaging modalities and data processing could also play a role.

This study also presents several limitations. First, in vivo cDTI data for only five subjects was considered. Although key results agree with other studies in the literature and our own ex vivo study and simulations, a larger cohort size in future studies will allow a strengthening of the current findings. As infarct size varied significantly across the imaged subjects, a larger cohort with varying infarct size will allow a better understanding of the dependence of diffusion tensor quantities on infarct size. Second, myocardial segmentation to determine the ground truth infarct, border, and remote regions based on LGE-PSIR data was carried out following the thresholding method described by Schelbert et al. [22]. Although previously adopted, uncertainties remain regarding the choice of the threshold values, which could result in small variations of the determined myocardial regions. Histological validation of the segmented remote, border, and infarct regions was not possible due to lack of histological data. Third, the difference in resolution, field of view (FOV), and cardiac phase between in vivo cDTI and LGE required interpolation and a non-rigid registration step. This might have led to the mislabeling of a small number of voxels in the boundary of the adjacent myocardial regions. Further work is required to quantify the uncertainty associated with non-rigid registration [45] along with the uncertainty due to the noise in the MRI images and observer error. Finally, regarding the diffusion simulation, a simplified geometric model was used to generate cellular structures, and simulations were carried out without considering cell permeability and intracellular diffusion. Given the higher sensitivity to extracellular diffusion of the adopted MRI sequence, we expect that the computed trends will remain representative even if a more realistic model is adopted. However, further work is needed to account for cell permeability, intracellular diffusion, multi-compartment cellular structures, and more realistic cell structures.

## 5. Conclusions

In vivo cDTI data that were acquired in swine subjects six to ten weeks post-myocardial infarction showed a decrease in fractional anisotropy and an increase in mean diffusivity and diffusion tensor eigenvalues in the infarct and border regions with respect to the remote myocardium. Across all subjects, the second (e_2_) and third (e_3_) diffusion tensor eigenvalues together with radial diffusivity (RD) showed the largest increase in the infarct and border regions compared to remote myocardium. As RD averages the changes in e_2_ and e_3_, it can be a potential biomarker to identify infarct regions without the use of contrast agents, and can provide additional information about microstructural changes occurring post-MI.

## Supplementary Material

In vivo cDTI, native T1, post-contrast T1, and LGE data for the analyzed swine subjects

## Figures and Tables

**Table 1.** Overall mean, median, and Q1–Q3 computed across all subjects for native T1, ECV, FA, MD, diffusion tensor eigenvalues, and RD in the infarct, border, and remote regions.

	Native T1 (ms)			ECV		
	Infarct	Border	Remote	Infarct	Border	Remote
Mean	1546	1434	1344	0.49	0.39	0.32
Median	1554	1389	1282	0.47	0.38	0.31
Q1, Q3	1363, 1700	1274, 1553	1210, 1442	0.36, 0.62	0.31, 0.46	0.27, 0.37
	FA			MD (1 × 10^−3^ mm^2^/s)		
	Infarct	Border	Remote	Infarct	Border	Remote
Mean	0.25	0.28	0.29	1.81	1.69	1.46
Median	0.23	0.27	0.29	1.84	1.68	1.46
Q1, Q3	0.18, 0.30	0.21, 0.33	0.23, 0.35	1.52, 2.12	1.45, 1.92	1.32, 1.60
	e_1_ (1 × 10^−3^ mm^2^/s)			e_2_ (1 × 10^−3^ mm^2^/s)		
	Infarct	Border	Remote	Infarct	Border	Remote
Mean	2.27	2.17	1.91	1.80	1.65	1.40
Median	2.28	2.14	1.94	1.83	1.65	1.38
Q1, Q3	1.98, 2.55	1.90, 2.39	1.74, 2.10	1.48, 2.11	1.37, 1.92	1.22, 1.58
	e_3_ (1 × 10^−3^ mm^2^/s)			RD (1 × 10^−3^ mm^2^/s)		
	Infarct	Border	Remote	Infarct	Border	Remote
Mean	1.38	1.25	1.06	1.59	1.45	1.23
Median	1.42	1.24	1.06	1.62	1.45	1.22
Q1, Q3	1.09, 1.71	1.01, 1.48	0.91, 1.23	1.29, 1.90	1.20, 1.69	1.08, 1.39

**Table 2.** Overall percentage change in median values and resulting *p*-values from non-parametric pairwise Kruskal–Wallis test for native T1, ECV, FA, MD, diffusion tensor eigenvalues, and RD between border–remote and infarct–remote regions across all subjects.

Native T1		ECV	
Border-Remote	Infarct-Remote	Border-Remote	Infarct-Remote
8.4%, *p* < 0.01	21.2%, *p* < 0.01	22.6%, *p* < 0.01	51.6%, *p* < 0.01
FA		MD	
Border-Remote	Infarct-Remote	Border-Remote	Infarct-Remote
−7.6%, *p* < 0.01	−19.8%, *p* < 0.01	14.7%, *p* < 0.01	26.1%, *p* < 0.01
e_1_		e_2_	
Border-Remote	Infarct-Remote	Border-Remote	Infarct-Remote
10.4%, *p* < 0.01	17.5%, *p* < 0.01	19.2%, *p* < 0.01	31.9%, *p* < 0.01
e_3_		RD	
Border-Remote	Infarct-Remote	Border-Remote	Infarct-Remote
16.1%, *p* < 0.01	33.4%, *p* < 0.01	18.0%, *p* < 0.01	32.3%, *p* < 0.01

**Table 3.** Median value percentage change and *p*-values resulting from non-parametric pairwise Kruskal–Wallis test between infarct–border, border–remote, and infarct–remote regions for each subject separately and for all measured quantities. Values reported in red correspond to *p*-values greater than or equal to 0.01 or isolated cases where percentage changes are opposite to the overall observed trends.

Sub	FA			MD		
	Infarct vs. Border	Border vs. Remote	Infarct vs. Remote	Infarct vs. Border	Border vs. Remote	Infarct vs. Remote
S1	−18.4%, *p* < 0.01	−24.0%, *p* < 0.01	−38.0%, *p* < 0.01	14.6%, *p* < 0.01	21.3%, *p* < 0.01	39.0%, *p* < 0.01
S2	−6.5%, *p* = 0.25	−2.7%, *p* < 0.01	−9.0%, *p* < 0.01	7.0%, *p* = 0.02	5.4%, *p* < 0.01	12.8%, *p* < 0.01
S3	−11.8%, *p* < 0.01	−11.1%, *p* < 0.01	−21.6%, *p* < 0.01	15.0%, *p* < 0.01	4.6%, *p* < 0.01	20.3%, *p* < 0.01
S4	−9.5%, *p* < 0.01	−5.7%, *p* = 0.1	−14.6%, *p* = 0.02	7.3%, *p* < 0.01	2.9%, *p* < 0.01	10.3%, *p* < 0.01
S5	−10.2%, *p* < 0.01	8.3%, *p* < 0.01	−2.7%, *p* < 0.01	7.1%, *p* < 0.01	6.0%, *p* < 0.01	13.5%, *p* < 0.01
Sub	e_1_			e_2_		
	Infarct vs. Border	Border vs. Remote	Infarct vs. Remote	Infarct vs. Border	Border vs. Remote	Infarct vs. Remote
S1	9.2%, *p* < 0.01	14.1%, *p* < 0.01	24.6%, *p* < 0.01	15.3%, *p* < 0.01	25.8%, *p* < 0.01	45.0%, *p* < 0.01
S2	3.7%, *p* = 0.22	3.2%, *p* < 0.01	7.0%, *p* < 0.01	13.7%, *p* < 0.01	6.9%, *p* < 0.01	21.5%, *p* < 0.01
S3	7.8%, *p* < 0.01	3.5%, *p* < 0.01	11.6%, *p* < 0.01	16.2%, *p* < 0.01	8.5%, *p* < 0.01	26.1%, *p* < 0.01
S4	7.1%, *p* < 0.01	−0.8%, *p* < 0.01	6.2%, *p* < 0.01	8.4%, *p* < 0.01	4.5%, *p* < 0.01	13.2%, *p* < 0.01
S5	4.1%, *p* < 0.01	7.8%, *p* < 0.01	12.2%, *p* < 0.01	6.7%, *p* < 0.01	6.7%, *p* < 0.01	13.8%, *p* < 0.01
Sub	e_3_			RD		
	Infarct vs. Border	Border vs. Remote	Infarct vs. Remote	Infarct vs. Border	Border vs. Remote	Infarct vs. Remote
S1	19.5%, *p* < 0.01	35.6%, *p* < 0.01	62.1%, *p* < 0.01	17.2%, *p* < 0.01	30.3%, *p* < 0.01	52.8%, *p* < 0.01
S2	5.1%, *p* = 0.19	8.3%, *p* < 0.01	13.8%, *p* < 0.01	10.0%, *p* = 0.01	8.7%, *p* < 0.01	19.6%, *p* < 0.01
S3	16.3%, *p* < 0.01	6.5%, *p* < 0.01	23.9%, *p* < 0.01	16.9%, *p* < 0.01	6.6%, *p* < 0.01	24.6%, *p* < 0.01
S4	10.4%, *p* < 0.01	5.2%, *p* < 0.01	16.2%, *p* < 0.01	7.7%, *p* < 0.01	4.2%, *p* < 0.01	12.2%, *p* < 0.01
S5	10.6%, *p* < 0.01	2.9%, *p* < 0.01	13.8%, *p* < 0.01	7.5%, *p* < 0.01	4.8%, *p* < 0.01	12.7%, *p* < 0.01
Sub	Native T1			ECV		
	Infarct vs. Border	Border vs. Remote	Infarct vs. Remote	Infarct vs. Border	Border vs. Remote	Infarct vs. Remote
S1	16.7%, *p* = 0.06	4.0%, *p* = 0.88	21.3%, *p* < 0.01	23.8%, *p* < 0.01	2.1%, *p* < 0.01	26.4%, *p* < 0.01
S2	18.7%, *p* = 0.66	14.1%, *p* < 0.01	35.4%, *p* < 0.01	61.7%, *p* < 0.01	29.2%, *p* < 0.01	108.9%, *p* < 0.01
S3	7.1%, *p* = 0.11	14.2%, *p* < 0.01	22.3%, *p* < 0.01	12.1%, *p* < 0.01	36.8%, *p* < 0.01	53.3%, *p* < 0.01
S4	2.0%, *p* = 0.3	4.5%, *p* < 0.01	6.6%, *p* = 0.018	10.1%, *p* < 0.01	11.3%, *p* < 0.01	22.6%, *p* < 0.01
S5	9.3%, *p* < 0.01	1.4%, *p* = 0.1	10.8%, *p* < 0.01	13.9%, *p* < 0.01	8.3%, *p* < 0.01	23.3%, *p* < 0.01

## Data Availability

The code to reconstruct the voxelwise diffusion tensors from cDTI data is freely available on GitHub at https://github.com/KMoulin/DiffusionRecon (Last accessed on 24 March 2022). The code to perform the diffusion simulations is also available on GitHub at https://github.com/KMoulin/DiffusionSimulation (Last accessed on 24 March 2022). In vivo cDTI, native T1, post-contrast T1, and LGE data for the analyzed swine subjects are provided as supplementary material.
